# Unusual acute neonatal mortality and sow agalactia linked with ergot alkaloid contamination of feed

**DOI:** 10.1186/s40813-019-0131-z

**Published:** 2019-11-06

**Authors:** Agnès Waret-Szkuta, Laurent Larraillet, Isabelle P. Oswald, Xavier Legrand, Philippe Guerre, Guy-Pierre Martineau

**Affiliations:** 1 IHAP, Université de Toulouse, INRA, ENVT, Toulouse, France; 2 Groupe Altitude, Aurillac, France; 3 Toxalim (Research Centre in Food Toxicology), Université de Toulouse, INRA, ENVT, INP-Purpan, UPS, 31027 Toulouse, France; 4 ENVT, Toulouse, France

**Keywords:** Agalactia, Ergot alkaloids, Wheat, Piglet mortality

## Abstract

**Background:**

An increase in the occurrence of ergot alkaloid contamination has been observed in Europe in recent years. The typical clinical signs of pig ergot poisoning are impaired growth, agalactia and, sometimes, gangrene. Opportunities for reporting exposure doses associated with clinical signs in animals under field conditions are rare.

**Case presentation:**

In a farrow-to-finish pig farm with 160 sows, excessive acute neonatal mortality was reported in association with a loss of appetite and agalactia in sows. A herd examination was conducted and a high rate of piglet loss and agalactia in 13 sows out of the most affected batch of 20 were confirmed. Necropsy showed piglets with empty stomachs and intestines, with apparently normal mucosa. Gestating and lactating sow diet samples, as well as a wheat sample, were sent for analysis following feed mill inspection and a hypothesis of mycotoxin contamination of self-prepared feed. Liquid chromatography with mass spectrometry in tandem revealed an amount of total ergot alkaloids in all of the samples ranging from 3.49 mg/kg (gestating diet) to 8.06 mg/kg (lactating diet). The contaminated feed was removed and the situation returned to normal 3 weeks later (following batch of sows).

**Conclusion:**

In the present case, the exposure of sows to 3.49 mg/kg ergot alkaloid for 10 to 15 days before the end of gestation and to 8.06 mg/kg ergot alkaloid over 3 to 4 days at the beginning of lactation - corresponding to a content of 10,146 mg of sclerotia/kg in the wheat of the diets- led to agalactia in 13 of 20 sows in a batch and to a high neonatal mortality rates for all litters. No clinical signs associated with vasoconstrictive effects were observed.

## Background

This report describes ergot alkaloid intoxication in a French farrow-to-finish pig farm of 160 sows, which led to high neonatal mortality and agalactia. Exposure of the affected sows was traced retrospectively because the clinical signs were acute and were coincident with the incorporation into the diet of a new batch of wheat.

Ergot is a parasitic fungus of the *Claviceps* genus which infests various grains, mainly rye, wheat and barley, while also infesting grasses. The *Claviceps* infestation is characterized by a dark mass of mycelium called sclerotia that produce toxic secondary metabolites: the ergot alkaloids (EAs) [1, 2]. More than 50 different EAs have been identified so far. The main EAs produced by *Claviceps* species are ergometrine, ergotamine, ergosine, ergocristine, ergocryptine and ergocornine [2]. Typical clinical signs of ergot poisoning reported in pigs are vasoconstriction, which may progress into gangrene, disruption of reproduction, abortion, agalactia and impaired growth [3, 4]. Although acute poisoning has become rare [2], EAs are a source of concern as they continue to be detected in cereals and cereal products in Europe and North America [5–7].

Depending on the country, regulations are based on the amount of sclerotia or the amount of total EA per kilogram of raw material or feed. In Europe, the European Commission fixed the maximum content at 1000 mg sclerotia /kg of feed stuff containing unground cereals [8]. Individual countries across the world have set different tolerance limits [9], and recent research suggests the limit deserves to be re-evaluated [2]. In Canada, the maximum allowable levels of EAs are defined per species and are between 4 and 6 mg/kg (ppm) in feed for pigs [9]. In general, 5–10 μg EAs /kg body weight (BW) represents the general threshold for all livestock, yet the European Food Safety Authority (EFSA) recommends doses as low as 0.6–1 μg EAs /kg BW to avoid their vasoconstrictive effects [3].

Different diagnostic tests are available for the detection of mycotoxins in grains and feeds, including thin-layer chromatography, near-infrared spectroscopy, high-pressure liquid chromatography (HPLC), enzyme-linked immunosorbent assay (ELISA) and liquid chromatography with mass spectrometry in tandem (LC/MS/MS) [4, 9]. Fluorescence has not been reported as reliable, and ELISA kits can be used for initial detection, but LC/MS/MS is considered the method of choice because it is highly specific and possibly can be used to identify multiple mycotoxins that may contaminate grains and feed at the same time [4].

Grain and feed samples should be representative of what is being consumed by the animals. However, due to the potential for mycotoxicosis to result in chronic complications, situations in which suspected grains of feed are still available are scarce, as are measurements of animal exposure in the field. Thus, the effect of EAs on pigs is mainly reported in experimental settings [2, 10, 11]. Case reports may enable fine-tuning of the regulatory limits for adequate protection.

## Case presentation

The case described occurred in a French 160 sow farrow-to-finish farm, managed in 7 batches of 20 sows every 3 weeks. Two buildings are on the premises. The first building contains the breeding area, the gestating sows and 2 rooms for weaned piglets. The second contains 3 farrowing rooms (2 for ten sows each and one for 20 sows) and 4 rooms for finishing pigs. Feed was homemade, except for the creep feed, and required the purchase of cereals when those harvested by the owner had been consumed (18 ha was cultivated for cereals). The feed was delivered manually to the farrowing rooms, which also contained individual drinkers. Sows entered the farrowing room 1 week before farrowing (on Thursday). They received a ‘gestating’ diet through Sunday and then a ‘lactating’ diet from Monday onwards (2 days before the expected farrowing date). The composition of the two diets covering the nutritional requirements of the pigs, is given in Table 1. The water used comes from an artesian borehole, is chlorinated (0.5 mg/L) and is regularly analysed. Performances were not recorded electronically, but the farmer reported weaning 10.5 piglets per litter on average and mean pre-weaning mortality was estimated to be around 17.3% the year before the visit.

**Table 1:** Gestating and lactating sow diet compositions and calculated net energy, crude protein and total lysine content

	Gestating diet	Lactating diet
Wheat	15.5%	48.51%
Barley	65.08%	25.99%
Soy	10.64%	12.51%
Extruded linseed	5.17%	4.81%
Minerals and vitamins	3.62%	4.33%
Bran	0%	3.85%
Net Energy (MJ)	9.6	9.7
Crude Protein (%)	14.2	15.0
Total Lysine (%)	0.64	0.66

Sudden excessive piglet mortality 24 to 48 h after birth occurred in association with a loss of appetite in the sows, which were prostrated and showed agalactia, leading the farmer to request a herd examination.

**Fig. 1:**
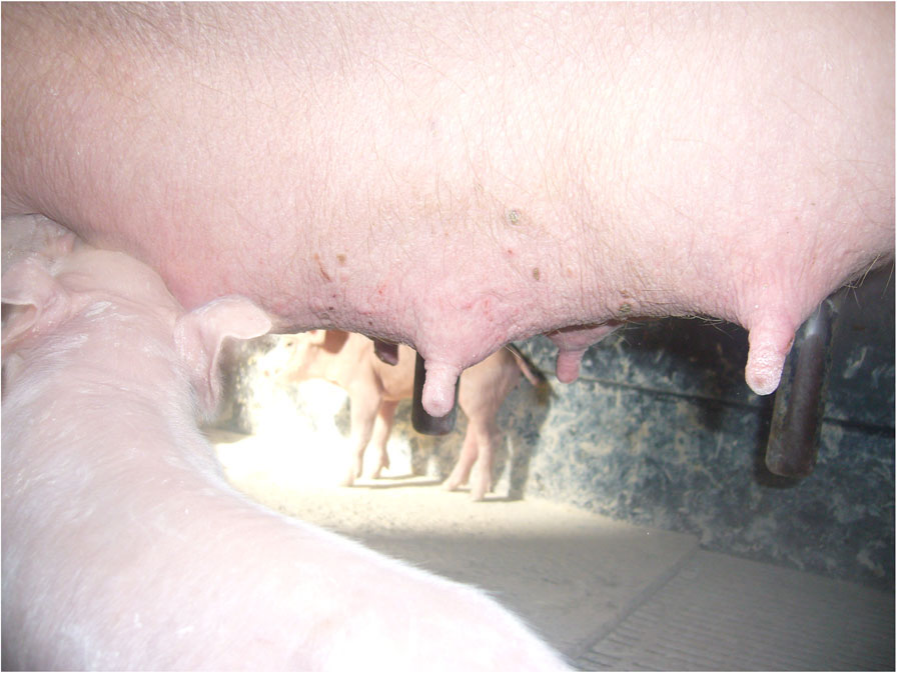
Udder with reduced development, sign of agalactia

The visit focused on the farrowing rooms as no noticeable clinical signs were observed in the breeding area or during gestation. The history revealed no particular problem related to the farrowing room’s environment. The room temperatures were set at 23 °C. In the first farrowing room, where the piglets had been born 12 to 48 h before our visit, all 20 sows were affected. Manually written information regarding each sow available in the room was compiled on a unique sheet. The mortality rate within the litters was on average 79%, ranging from 23% (3 piglets out of 13) to 100% (16 piglets out of 16). In litters less affected, the remaining piglets were emaciated but still alive. Sows were either lying ventrally with no udder access for the piglets or showed agalactia (Fig. 1) with no proper maternal behaviour.

The sows showed loss of appetite (3 unemptied feeders out of 20, of which one was full) and 50% seemed abnormally passive when feed was distributed. Their backfat depth was not measured, and although the condition seemed sub-optimal, no shoulder ulceration was noticed. Rectal temperatures from the sows that had not finished their meals were slightly below what was expected (38.3 °C and 38.6 °C vs 39.1 °C). Sow faeces were normal. The environment seemed a little cool for the piglets, as some piglets were piled.

In the second farrowing room, piglets were 3 weeks older. Piglet mortality rates were on average 24%, ranging from 0 to 62.5% (6 weaned piglets out of 16 born alive). The mean prolificity was 13.7, the mean stillborn rate was 0.75 (range from 0 to 4), and the mean number of weaned piglets per litter was 9.7, which was low compared to the usual performances on the farm.

Seven piglets from seven different litters of the first farrowing room were necropsied. One showed haemorrhage on the right kidney and intestine, probably due to crushing. The stomach was empty with apparently normal mucosa. No lesions were observed in the 6 other piglets. They had empty stomachs and intestines with apparently normal mucosa.

Careful examination of the feeding system was conducted, and multiple samples of self-prepared ground feed for gestating and lactating sows were taken, as well as wheat samples, because of a suspicion of ergot intoxication. The multiple samples taken from each of the feeds and wheat were pooled, thoroughly mixed and subsampled to ensure the representativeness of each final sample. Silos were regularly emptied, and wheat began to be distributed 15 days before the first farrowing of a sow in the most problematic farrowing room.

**Fig. 2:**
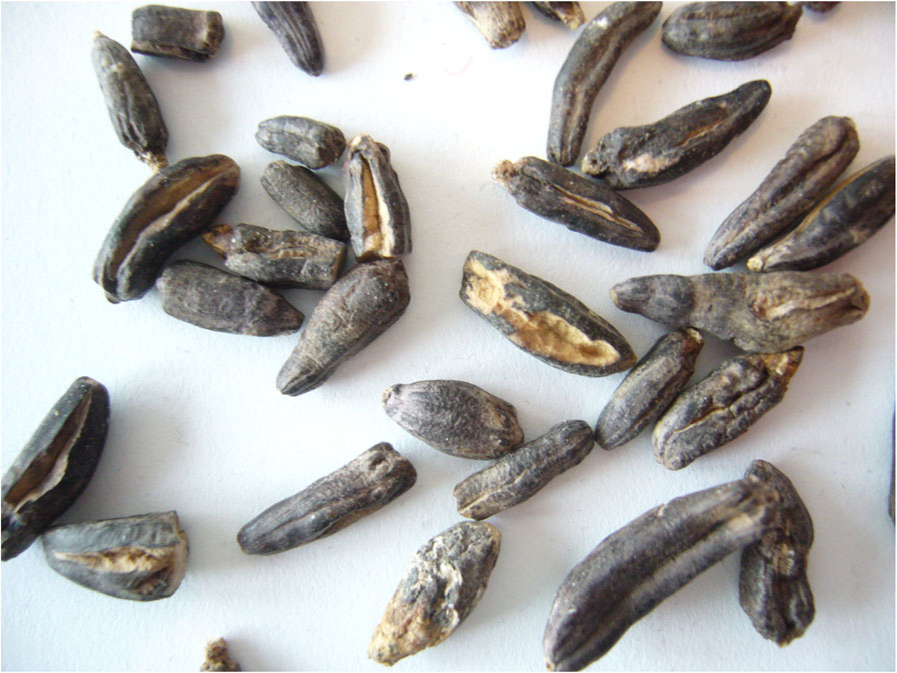
Wheat ergot sclerotia

Observation of the wheat sample revealed long and black grains: ergot sclerotia (Fig. 2).

Mycotoxin analysis by liquid chromatography with mass spectrometry in tandem was performed in a laboratory located in Brittany. Values obtained for mycotoxins other than the EAs (Trichothecenes, DON, Aflatoxins, Fumonisins, Zearalenone) were in the normal range of values [1, 4] and are not presented here (Table 2).

**Table 2:** Laboratory results for the main ergot alkaloids as determined by the LC-MS/MS method

Sample	Results (mg/kg)		Maximum recommended level of ergot alkaloids or recommended practical limits (mg/kg) [9]
Feed 1 (Lactation feed)	Ergocornine	0.395	Low: 0.5; Moderate: 1; High: 2 4–6 (Canada)
	Ergocristine	2.22	
	Ergocryptine	0.690	
	Ergometrine	0.285	
	Ergosine	1.56	
	Ergotamine	2.91	
	Total	8.06	
Feed 2 (Gestation feed)	Ergocornine	0.295	
	Ergocristine	0.845	
	Ergocryptine	0.600	
	Ergometrine	0.145	
	Ergosine	0.580	
	Ergotamine	1.02	
	Total	3.49	
Wheat	Ergocornine	0.670	
	Ergocristine	3.41	
	Ergocryptine	1.39	
	Ergometrine	0.760	
	Ergosine	3.10	
	Ergotamine	6.73	
	Total	16.06	

Levels of ergot alkaloids were summed and are presented in Table 2 for each of the feeds and the wheat. Amount of total ergot alkaloids ranged from 3.49 mg/kg in the gestation feed to 8.06 mg/kg in the lactation feed. It was of 16.06 mg/kg in the wheat. For the batch of sows in the first farrowing room, contaminated gestation diet was fed for approximately 2 weeks and the lactation diet for 3 to 4 days. In the second farrowing room, sows likely received 2 weeks of lactating diet and normal gestation diet.

The wheat was also visually inspected. The laboratory returned a result of 10,146 mg of sclerotia/kg, which corresponded to 4922 mg of sclerotia/kg in the lactation diet.

The farmer received recommendations to (i) remove wheat from the diets and to provide milk replacer or creep feed to the remaining piglets; (ii) increase the room temperature in the farrowing room with the youngest piglets; and (iii) distribute sorbitol to sows to stimulate appetite, while also adding clay (2%) to the feed of the animals during the following few weeks. Overall, 76 and 25% of the piglets died from the first and the second farrowing rooms, respectively. Some piglets showed diarrhoea but the situation returned to normal after 3 weeks (next batch of sows). The impact on the animal’s growth could not be determined as no electronic records were kept. The wheat was kept in the feed bin until evaluation for insurance. The bin was then completely emptied, washed and fumigated. Investigation on the wheat origin revealed it had been refused for seed selection, which explained its low price. It had been analysed at the vendor level in terms of maximum acceptable level of ergot contamination, but information was lost and did not reach the farmer before incorporation into the diet.

## Discussion

Ergot intoxication in pigs is rarely reported in the field. However, EAs are a source of concern in Europe and North America for animals and also for humans because they continue to be detected in cereals and cereal products [5, 6]. Issues still exist with recommendations of safe levels of ergot in feeds, and the EFSA determined that new validated methods are still required to quantify ergot alkaloids in feed material to provide more reliable regulatory limits for each individual alkaloid. Allowable levels of ergot contamination in cereal grains and feed are variable depending on the regions of the world, and acceptable exposure levels depend on the species considered [9]. Recent experimental studies suggest that regulatory limits should be revised, and field reports could contribute to this revision [2]. Indeed, as the clinical form was acute in this case, we had the chance to sample the grain and feed consumed by the animals. Exposure estimations that reflect the timing of the exposure are important, as is the specific susceptibility of the last third of gestation [4].

The physical appearance of grain is not an accurate indicator for the presence of mycotoxins, and the correlation between spore counts and/or the fungal growth and concentration of mycotoxins is reported to be low [4]. However, our observations could not corroborate this claim, as the quantity of sclerotia was associated with the amount of ergot alkaloids. This association may have occurred by chance, although it may also question the trend to prefer analytical methods.

Indeed, the effect of the major toxic alkaloids that were dosed is reported to be additive. Sow agalactia is the result of stimulation of the D2-dopamine receptors, which leads to prolactin suppression in pregnant sows fed ergot sclerotia [12]; piglets can be born healthy but starve because of agalactia and die from hypoglycaemia. Kopinsky (2008) [11] showed that *Claviceps africana* (sorghum) ergot sclerotia fed to sows and forming up to 1.5% of the diet (equivalent to 7 mg/kg ergot alkaloids) 6–10 days prior to parturition caused agalactia, with 87% of the piglets dying. Those results are similar to our observations, although a direct comparison is problematic as *Claviceps purpurea* does not produce exactly the same alkaloids. The value found for the lactation diet for the quantity of sclerotia per kilogram of lactation feed was 5 times the regulated maximum European level of 1000 mg/kg [8].

In the present case study, we did not observe any vasoconstriction. This might be at least partly due to the short exposure of the animals. Indeed, in the literature, vasoconstriction often occurs upon exposure to contaminated feed for a longer period, usually several weeks [2]. Nevertheless, hematology and blood chemistry would have been interesting to complete the investigation and assess the potential changes not visible solely by clinical observation such as a reduction of the percentage (%) of neutrophils, an increase of the % of lymphocytes or a reduction in the level of creatine kinase [2]. Management of the clinical case may also have been improved by the necropsy of more animals. Indeed, only 7 piglets were necropsied, one from each sow showing agalactia. This represents only 3.2% of the piglet losses (*n* = 218) after the deduction of stillbirths, and may not be representative. If the necropsy results were indeed consistent with our hypothesis, we might have introduced cognitive bias when making a diagnostic decision. In the literature, this is described as setting an initial hypothesis and ignoring or not looking for salient data [13]. However, the situation returned to normal in the next batches after the contaminated wheat was removed, confirming our hypothesis that the problem was linked to the feed.

Milk production, which is non-responsive to oxytocin when ergot intoxication occurs [14], is reported to return 3–7 days after the feed has been changed. In the interim, the use of supplemental nutrition and milk replacers, which we did, is recommended to save the piglets. This change could have begun by the owner before the visit, which was not the case and reflects poor management. Clay addition was recommended as a mycotoxin binder. However, solely physical methods of cleaning to remove ergot bodies have been reported to be effective in the literature [9].

## Conclusion

The exposure of sows to 3.49 mg/kg ergot alkaloid for 10 to 15 days before the end of gestation and to 8.06 mg/kg ergot alkaloid over 3 to 4 days at the beginning of lactation - corresponding to a content of 10,146 mg of sclerotia/kg in the wheat of the diets- led to agalactia in 13 of 20 sows of a batch and to a high neonatal mortality rate for all litters. Up to 76% of the piglets died in the most affected farrowing room. Wheat was removed from the diet and the situation returned to normal after 3 weeks (next batch of sows). Although acute poisoning has become rare it remains possible. Reporting exposure doses associated with clinical signs and blood parameters may enable fine-tuning of the regulatory limits for adequate protection.

## Data Availability

Data sharing not applicable to this article as no datasets were generated or analysed during the current study.

## Abbreviation

EAs: Ergot Alkaloids
